# Snail communities increase submerged macrophyte growth by grazing epiphytic algae and phytoplankton in a mesocosm experiment

**DOI:** 10.1002/ece3.8615

**Published:** 2022-02-14

**Authors:** Tian Lv, Xin Guan, Shufeng Fan, Chen Han, Zhongyao Gao, Chunhua Liu

**Affiliations:** ^1^ 12390 The National Field Station of Freshwater Ecosystem of Liangzi Lake College of Life Science Wuhan University Wuhan China

**Keywords:** coexistence, exotic macrophyte, grazing preferences, snail–macrophyte–algae relationship

## Abstract

The relationships between producers (e.g., macrophytes, phytoplankton and epiphytic algae) and snails play an important role in maintaining the function and stability of shallow ecosystems. Complex relationships exist among macrophytes, epiphytic algae, phytoplankton, and snails. We studied the effects of snail communities (consisting of *Radix swinhoei*, *Hippeutis cantori*, *Bellamya aeruginosa*, and *Parafossarulus striatulus*) on the biomass of phytoplankton and epiphytic algae as well as on the growth of three species of submerged macrophytes (*Hydrilla verticillata*, *Vallisneria natans*, and one exotic submerged plant, *Elodea nuttallii*) in a 90‐day outdoor mesocosm experiment conducted on the shore of subtropical Lake Liangzihu, China. A structural equation model showed that the snail communities affected the submerged macrophytes by grazing phytoplankton and epiphytic algae (reduction in phytoplankton Chl‐*a* and epiphytic algal abundance), enhancing the biomass of submerged macrophytes. Highly branched macrophytes with high surfaces and morphologies and many microhabitats supported the most snails and epiphytic algae (the biomass of the snail communities and epiphytic algae on *H*. *verticillata* was greater than that on *V*. *natans*), and snails preferred to feed on native plants. Competition drove the snails to change their grazing preferences to achieve coexistence.

## Graphical abstract

We found that the snail communities reducing the biomass of phytoplankton and epiphytic algae indirect then enhanced the growth of the submerged macrophytes. The macrophyte with complex architecture supported more snail and epiphytic algae, and snails preferred to feed on native plants. Competition drove snails change the grazing preferences to achieve coexistence.

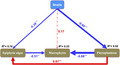

## 1 INTRODUCTION

Submerged macrophytes, phytoplankton, epiphytic algae, and aquatic snails are important taxa in freshwater ecosystems and are widely distributed in various water bodies (Carpenter & Lodge, 1986; Underwood et al., 1992; Zhu et al., 2013). The relationship between producers (e.g., macrophytes, phytoplankton and epiphytic algae) and snails is important in maintaining the function and stability of shallow‐water ecosystems (Jeppesen et al., 1998; Kuiper et al., 2017; Scheffer, 1999; Underwood et al., 1992; Yang et al., 2020). Submerged macrophytes inhibit epiphytic algae and phytoplankton through the reduction of nutrients, allelopathy and shading (Casartelli & Ferragut, 2018; Hilta & Grossb, 2008; Mohamed & Shehri, 2010; Sand‐Jensen & Borum, 1991). Submerged macrophytes also represent an important food source and critical habitats for aquatic animals (Brix, 1994; Zhi et al., 2020). Heterogeneity in macrophytes with distinct structures can affect epiphytic algae (Hao et al., 2017; Santos et al., 2013) and therefore food availability for the invertebrate community (Mason & Underwood, 2010; Thomaz et al., 2008). In addition, the relationship between epiphytic algae, phytoplankton, snails, and macrophytes is affected by evolutionary history (Keane & Crawley, 2002; Ficetola, 2020). Snail and algae have generally adapted to the defence strategies of native plants over long‐term coevolution, while they can be naive to the defence strategies of exotic plants (Keane & Crawley, 2002; Xiong et al., 2008).

Phytoplankton and epiphytic algae are the main primary producers that compete with macrophytes for light, nutrients, and space (Arthaud et al., 2012; Phillips et al., 2016; Song et al., 2017). Phytoplankton and epiphytic algae are also the main food sources of aquatic animals in freshwater ecosystems, such as fish, shrimp, snails, oligochaetes, mayflies, and chironomids (Asch et al., 2019; Chen et al., 2020; Guasch et al., 2016). Epiphytic algae and phytoplankton are considered the key factors causing the transformation between clear and turbid states in shallow aquatic ecosystems (Phillips et al., 2016; Qin et al., 2013). In the turbid state, the establishment and growth of submerged macrophytes may be restricted due to light attenuation induced by high phytoplankton and epiphytic algae biomass (Arthaud et al., 2012; Hidding et al., 2016), while high grazing pressure from predators reduces the biomass of phytoplankton and epiphytic algae, which then increases light availability and promotes macrophyte growth in the clear water state (Hilt, 2015; Sánchez et al., 2010).

Freshwater snails filter feed on phytoplankton in the water, scrape organic detritus, and periphyton from surfaces and sometimes also feed on macrophytes (Cao et al., 2014; Li et al., 2009; Yang et al., 2020). Most of freshwater snails are scrapers, and others are collector‐filterers (Mo et al., 2017). Scrapers consume mainly epiphytic algae, but their diet also includes detritus and aquatic plants (Li et al., 2009). Collector‐filterers use gills to filter suspended algae from the water column (Yang et al., 2020). Snail–algae interactions may thus be of great importance for submerged macrophytes. The grazing of epiphytic algae and phytoplankton increases the growth rates of macrophytes, potentially by reducing competition for light and/or nutrients (Brönmark, 1989; Yang et al., 2020). The above phenomenon is called a snail–macrophyte mutualistic interaction (Carpenter & Lodge, 1986; Li et al., 2007). Macrophytes, however, are also grazed by snails, which may have a significant impact on macrophyte growth (Elger & Lemoine, 2005; Li et al., 2009; Xiong et al., 2008). For example, *Radix swinhoei*, a member of Lymnaeidae, not only scrapes organic detritus and periphyton from the surface but also feeds on macrophytes (Li et al., 2006). Therefore, the relationship between snails and macrophytes remains unclear. Snails also exhibit complex and plastic behaviors when coexisting with other snails (Lombardo & Cooke, 2004). Overlapping food sources of freshwater snails may lead to competition (Holomuzki & Hemphill, 1996), and changes in resource utilization by competing snail species may impact food web dynamics and community assembly (Estebenet et al., 2002). However, studies of interspecific interactions among freshwater snails are uncommon (Dubart et al., 2019; Turner et al., 2007).

The ecological mechanisms by which snail communities affect macrophyte growth, phytoplankton biomass, epiphytic algal communities, and nutrient cycling and transformation are unclear. We hypothesized that snail grazing on both epiphytic algae and phytoplankton can indirectly improve the growth of submerged macrophytes. We further hypothesized that competition drives snails to change their grazing preferences to achieve coexistence, which leads snail communities toward maximal resource utilization. To test our hypotheses, we conducted an outdoor mesocosm experiment to elucidate the effects of snail communities on aquatic ecosystems. We studied the effects of snail communities (consisting of *Radix swinhoei*, *Hippeutis cantori*, *Bellamya aeruginosa*, and *Parafossarulus striatulus*) on the biomass of phytoplankton and epiphytic algae as well as on the growth of three species of submerged macrophytes (*Hydrilla verticillata*, *Vallisneria natans* and one exotic submerged plant, *Elodea nuttallii*) in a 90‐day outdoor mesocosm experiment conducted on the shore of subtropical Lake Liangzihu, China.

## 2 MATERIAL AND METHODS

### 2.1 Experimental design

An outdoor mesocosm experiment was conducted at the National Field Station of the Freshwater Ecosystem of Liangzi Lake (hereinafter referred to as Liangzi Lake Station), Hubei Province, China. A two‐way factorial experiment was carried out with three species of submerged macrophytes (*Hydrilla verticillata*, *Vallisneria natans*, or *Elodea nuttallii*, the three species macrophytes were planted in their respective experimental vessels) and two grazing treatments (four species snails present or snail absent), with six replicates for each treatment, resulting in a total of 36 aquariums. The study began on August 21, 2017, and ended on December 21, 2017. (The timing of the experimental harvest based on the growth of the three macrophytes: 90 days is the three macrophytes just ended their vegetation period, and the snails completed 3–4 life histories.) At the time of experimental harvest (December 21, 2017), water physical and chemical characteristics, epiphytic algae, macrophytes, and snails were measured.

Thirty‐six glass fibre‐reinforced polymer (GFRP) aquariums (inner diameter: 40 cm, height: 70 cm, Figure S1 ) were placed on a cement platform (50 m long, 20 m wide, Figure S1). The sediment used in our experiment was collected from Liangzi Lake. To ensure homogeneity and remove benthic animals (especially snails) before the experiment began, the sediment was air dried under natural conditions, ground, sieved (0.6 mm mesh size), and mixed before being added to the aquarium. To each aquarium, we added 10 cm of sediment (nitrogen content: 0.56 ± 0.05 mg g^−1^, phosphorus content: 1.63 ± 0.02 mg·g^−1^, organic matter content: 0.068 ± 0.003 mg g^−1^; all values are means ± SD). We subsequently added 70 L of groundwater (total nitrogen (TN): 0.52 mg L^−1^ and total phosphorus (TP): 0.03 mg L^−1^).

*Hydrilla verticillata* and *V*. *natans* are the dominant macrophytes in Liangzi Lake (Wang et al., 2019; Xu et al., 2018), and *E*. *nuttallii* is an invasive species in China (Xiong et al., 2008). On August 21 2017, 72 specimens of the submerged macrophytes *H*. *verticillata*, *V*. *natans*, and *E*. *nuttallii* were collected from a homogeneous population in the nursery ponds of the Liangzi Lake Station. All plants were carefully washed to remove snail eggs and periphyton, and six shoots of each macrophyte species were planted in each aquarium. Before planting in an aquarium, each macrophyte species selected had standardized biomass and length (*H*. *verticillata*: 0.53 ± 0.12 g and 20 ± 2 cm, respectively; *V*. *natans*: 1.08 ± 0.99 g and 15 ± 2 cm; *E*. *nuttallii*: 0.41 ± 0.09 g and 18 ± 2 cm; each value represents the mean ± SD).

On September 21, 2017, a large number of vigorous and sexually mature snails *Radix swinhoei*, *Hippeutis cantori*, *Bellamya aeruginosa*, and *Parafossarulus striatulus* were collected from the macrophyte plants growing in the nursery ponds of Liangzi Lake Station. The snails were kept without food for 24 h before being added to the aquarium (Xiong et al., 2008). Subsequently, we selected 360 individuals of four species snails of homogeneous size and age. Of these species, *R*. *swinhoei* and *H*. *cantori* are hermaphroditic and undergo allogeneic fertilization, while *B. aeruginosa* and *P*. *striatulus* are dioecious (Li et al., 2009). Therefore, the ratio of females to males that we selected for *B. aeruginosa* and *P*. *striatulus* in this study was 1 to 1. After the submerged plants had grown for over one month (on September 21), 80 individuals (20 individuals of each snail species) were added to each aquarium, which was then covered by a nylon net (1.0 mm mesh size) to prevent snail escape. The fresh mass of the snail species was as follows: *R*. *swinhoei*: 0.38 ± 0.04 g ind.^−1^, *H*. *cantori*: 0.04 ± 0.01 g·ind.^−1^, *B. aeruginosa*: 2.33 ± 0.15 g ind.^−1^, and *P*. *striatulus*: 0.16 ± 0.01 g ind.^−1^. The water level of the aquariums regularly topped up to the initial level with pure water during the experiment.

### 2.2 Sampling and analysis

#### 2.2.1 Water physical and chemical characteristics

For each aquarium, the water temperature (*T*), dissolved oxygen (DO), conductivity (Cond), and pH of the water were measured with a portable water quality monitor (PROPLUS, YSI), and chlorophyll a (Chl‐*a*) was measured with a handheld chlorophyll fluorometer probe (HYDROLAB DS5, HACH) in the field tests. Turbidity (Turb) was measured with a chromometer (DR900, HACH). We collected 1 L water samples from each aquarium with depth integration (under water 30 cm) for chemical analysis and stored them on ice. Then, TN, TP, and ammonia nitrogen (NH_3_‐N) were analyzed with a flow injection analyser (QC8500, LACHAT). Chemical oxygen demand (COD) was analyzed with a digestion solution for each corresponding parameter and landscape photometry (DR900, HACH).

#### 2.2.2 Epiphytic algae

Fifty leaves of *H*. *verticillata*, 50 leaves of *E*. *nuttallii*, and five leaves of *V*. *natans* were carefully selected to ensure uniformity in growth state and size before placing each into a wide‐mouth plastic bottle with 200 ml of pure water in the respective aquarium. Periphyton were removed with a banister brush in water (Foerster & Schlichting, 1965) and preserved in a well‐labeled plastic container, with 2 ml of Lugol's solution to fix them. The area of selected leaves was measured with an area meter (LI‐3100C, LI‐COR). The epiphytic algae sample was centrifuged at 1788.8 *g* for 10 min, and the supernatant was discarded. Then, the volume was adjusted to 30 ml and mixed. The number and species of epiphytic algae were counted using a counting plate at 400× under an optical microscope. For each sample, 50 microscopic fields of vision were examined and counted. (Effiong & Inyang, 2015; Hu & Wei, 2006; Qian et al., 2015). Species richness (S) of each sample was quantified as the number of species in the sample, and the abundance (*N*, cells) was the total number of individual quantities and calculated using the following formula:

$$N=\frac{\text{total}\;\text{leaf}\;\text{number}}{\text{selected}\;\text{leaf}\;\text{number}}\times \sum_{i=1}^{S}n_{i}$$

where *n_i_* is the quantity of species *i* and *S* is the number of species.

#### 2.2.3 Macrophytes

The macrophyte samples were carefully washed with distilled water at least three times. Then, the number of leaves in each sample was quantified (including the selected leaf for area measurement and algae collection). All samples were then dried to a constant weight in a drying oven at 60°C. The dry weight of biomass of the submerged macrophytes was determined using an electronic scale.

#### 2.2.4 Snail

All snail individuals (adults and offspring) were collected from the aquariums and the quantity and fresh mass were determined. Before weighing, the snails were drained and allowed to dry on absorbent paper for 5 min (wiping the surface of snails and letting the liquid drain from their body) and then gently blotting until the surface was dry to ensure consistency among the samples (Yang et al., 2020).

### 2.3 Data analyses

We used two‐way ANOVA to test for the effects of macrophytes, grazing treatment, and their interaction on the environmental factors (i.e., *T*, DO, Cond, pH, TN, TP, NH_3_‐N, and COD), followed by the least significant difference (LSD) post hoc test. The relative growth rate (RGR) of the macrophytes and snails was calculated according to the equation RGR (mg g^−1^ day^−1^) = 1000·ln (*W*_f_/*W*_i_)/days, where *W*_f_ (g) and *W*_i_ (g) are the average final and initial mass of the snails or macrophytes in each aquarium, respectively, in grams (Gu et al.,^2018^). The effects of macrophyte species, grazing treatment, and their interaction on the biomass and RGR of macrophytes were determined using two‐way ANOVA with post hoc LSD tests for multiple comparisons. The data describing the characteristics of snails (i.e., number, biomass, and RGR, at the total species level) from macrophytes were evaluated using one‐way ANOVA with post hoc LSD tests for multiple comparisons. Two‐way ANOVA was used to assess macrophyte and snail species effects on snail characteristics (i.e., number and biomass at the species level), and post hoc LSD tests were conducted for multiple comparisons. The effects of macrophytes, grazing treatment, and their interaction on phytoplankton biomass (Chl‐*a* concentrations and Chl‐*a* concentrations in the water were used as surrogates for phytoplankton biomass.) and epiphytic algae numeral traits (richness and abundance) were investigated using two‐way ANOVA with post hoc LSD tests for multiple comparisons.

To determine the relative importance of the direct versus indirect effects of snails on macrophytes, we built a structural equation model (SEM, Table S3) (Oberski et al., 2014) including total RGR of snails, epiphytic algae biomass (abundance, abundance was the total number of epiphytic algae and which was used as a surrogate for epiphytic algae biomass), phytoplankton biomass (Chl‐*a*), and RGR of macrophytes (data including treatments of snail‐present and snail‐absent, total of 36 samples). Our hypothesis assumes that snail composition is influenced by measured environmental variables. Prior to the main statistical analyses, we disproved the correlation of environmental variables (data including snail‐present treatments, total of 18 samples). By performing a principal component analysis (PCA) to eliminate collinearity of nutrient factors, the nutrient factors (i.e., TN, TP, NH_3_‐N, and COD) were reduced to the first principal component (proportion variance of PC1 = 0.97, Table S2) as an explanatory variable reflecting nutrients (Nutrient). Redundancy analysis (RDA) was carried out as follows: imported data included snail biomass and environmental variables (i.e., macrophyte biomass, epiphytic algae abundance, phytoplankton Chl‐*a* content, water temperature, dissolved oxygen, and nutrient), Hellinger transformation with downweighting of rare species, and biplot scaling focused on interspecies distances (Table S4A,B). A Monte‐Carlo permutation test was used (reduced model, 499 permutations) to determine the relative weight of environmental factors on snail composition (Table S4C,D). The Spearman rank correlation coefficient was used to assess the correlation between four species of snails (Hellinger transformed biomass) and environmental factors.

To ensure that the data conformed to the assumptions of a normal distribution and homogeneity of variance, some parameters were log10 transformed before performing ANOVA, SEM, PCA, or RDA. Statistical analyses were performed using r version 3.6.3 with the packages agricolae (Mendiburu, 2018), vegan (Oksanen et al., 2018) and lavaan (Oberski et al., 2014), and the significance level was set to *p* < .05.

## 3 RESULTS

### 3.1 Variations in water environmental factors

During the experiment, the concentrations of DO, Cond, Turb, TN, TP, NH_3_‐N, and COD were notably affected by both submerged macrophyte species and snail presence (*p* < .05, Table 1). The presence of snails consistently led to significantly lowered concentrations of nutrients (i.e., TN, TP, NH_3_‐N, and COD) in the water associated with the three macrophyte species (*p* < .001, Table 2). The concentrations of nutrients (i.e., TN, TP, NH_3_‐N, and COD) in the presence of *H*. *verticillata* were lowest when snails were both present and absent (Table 2). Water temperature was not affected by submerged macrophyte species or snail presence (Table 1, *p* = 1.000). pH was affected only by submerged macrophyte species (Table 1, *p* = .006). There were significant interactions between macrophyte species and snail presence for DO and Cond (*p* < .05, Table 1) but not for Turb, TN, TP, NH_3_‐N, or COD (*p* > .05, Table 1).

**TABLE 1:** Effect of macrophyte species and snail grazing on the water environmental factors during the experiment using two‐way ANOVA (values in bold are below the significance level of .05)

	Macrophyte	Snail grazing	Macrophyte × snail grazing
*T*			
df	2	1	2
*F*	0.00	0.00	0.00
*p*	>.999	>.999	>.999
DO			
df	2	1	2
*F*	287.89	138.46	4.53
*p*	**<.001**	**<.001**	.**019**
Cond			
df	2	1	2
*F*	366.75	8.33	4.08
*p*	**<.001**	.**007**	.**027**
pH			
df	2	1	2
*F*	6.15	1.63	0.01
*p*	.**006**	.211	.99
Turb			
df	2	1	2
*F*	35.06	18.99	2.6
*p*	**<.001**	**<.001**	.09
TN			
df	2	1	2
*F*	51.3	46.93	0.73
*p*	**<.001**	**<.001**	.496
TP			
df	2	1	2
*F*	71.59	30.1	0.34
*p*	**<.001**	**<.001**	.715
NH_3_‐N			
df	2	1	2
*F*	27.65	30.12	0.87
*p*	**<.001**	**<.001**	.43
COD			
df	2	1	2
*F*	21.19	14.56	0.06
*p*	**<.001**	**<.001**	.945

Abbreviations: COD, chemical oxygen demand; Cond, conductivity; Do, dissolved oxygen; NH_3_‐N, ammonia nitrogen; *T*, temperature; TN, total nitrogen; TP, total phosphorus; Turb, turbidity.

**TABLE 2:** Comparison of environmental factors associated with macrophyte and snail grazing treatments during the experiment on the basis of water temperature (*T*), dissolved oxygen (DO), turbidity, total nitrogen (TN), total phosphorus (TP), ammonia nitrogen (NH_3_‐N), and chemical oxygen demand (COD). Values represent the mean ± SD; means with the different letters are significantly different at *p* < .05 (LSD test)

	Snail‐absent			Snail‐present		
	*E. nattalii*	*V. natans*	*H. verticillata*	*E. nattalii*	*V. natans*	*H. verticillata*
*T* (°C)	16.2 ± 0.06a	16.2 ± 0.063a	16.2 ± 0.06a	16.2 ± 0.06a	16.2 ± 0.06a	16.2 ± 0.06a
DO (mg L^−1^)	8.88 ± 0.01d	8.94 ± 0.02c	9.02 ± 0.01b	8.92 ± 0.01c	9.01 ± 0.02b	9.11 ± 0.02a
Conductivity (μS cm^−1^)	110.6 ± 0.1a	110.2 ± 0.1b	109.5 ± 0.2cd	110.6 ± 0.1a	110.2 ± 0.1b	109.2 ± 0.1d
pH	7.74 ± 0.01a	7.74 ± 0.11a	7.66 ± 0.02a	7.77 ± 0.06a	7.77 ± 0.06a	7.68 ± 0.08a
Turbidity (NTU)	6.62 ± 0.11ab	6.71 ± 0.12a	5.65 ± 0.71c	6.14 ± 0.61bc	6.32 ± 0.07ab	4.46 ± 0.67d
TN (mg L^−1^)	0.38 ± 0.007a	0.37 ± 0.014a	0.34 ± 0.008c	0.36 ± 0.011b	0.35 ± 0.008bc	0.31 ± 0.012d
TP (mg L^−1^)	0.018 ± 0.002a	0.017 ± 0.001b	0.014 ± 0.001c	0.017 ± 0.001b	0.015 ± 0.001c	0.011 ± 0.001d
NH_3_‐N (mg L^−1^)	0.011 ± 0.001a	0.011 ± 0.001a	0.009 ± 0.001c	0.01 ± 0.001b	0.009 ± 0.001bc	0.007 ± 0.001d
COD (mg L^−1^)	6.5 ± 0.84a	6.2 ± 0.75ab	4.8 ± 0.41d	5.7 ± 0.82bc	5.3 ± 0.52cd	3.8 ± 0.75e

### 3.2 Macrophyte

**FIGURE 1:**
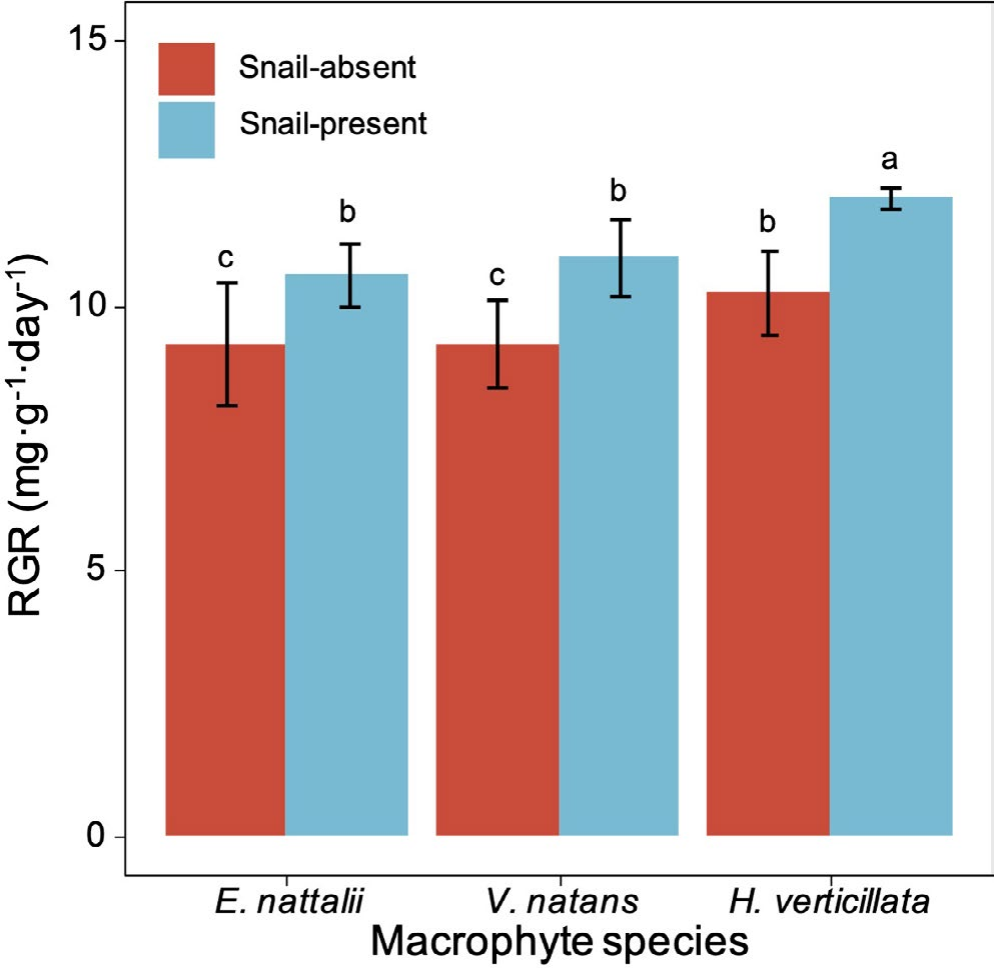
Comparison of the relative growth rates (RGRs) of *E*. *nuttallii*, *H*. *verticillata*, and *V*. *natans* in the different snail grazing treatments during the experiment. Values represent the mean ± SD, and means with different letters are significantly different at *p* < .05 (LSD test)

The RGRs of the three species of macrophytes were markedly affected by their species and snail presence (Table 3, *p* < .05), but the interactions between these two variables were nonsignificant. Snails significantly led to an increase in the RGR of the *H*. *verticillata*, *V*. *natans*, and *E*. *nuttallii*, (Figure 1), with *H*. *verticillata* having the greatest RGR among the three submerged macrophyte species when snails were present (Figure 1).

**TABLE 3:** Effects of snail grazing on the relative growth rate (RGR) of three submerged macrophytes during the experiment using two‐way ANOVA (values in bold are below the significance level of .05)

	RGR		
	df	*F*	*p*
Macrophyte	2	8.65	.**001**
Snail grazing	1	39.29	**<.001**
Macrophyte × snail grazing	2	0.29	.75

### 3.3 Snails

**FIGURE 2:**
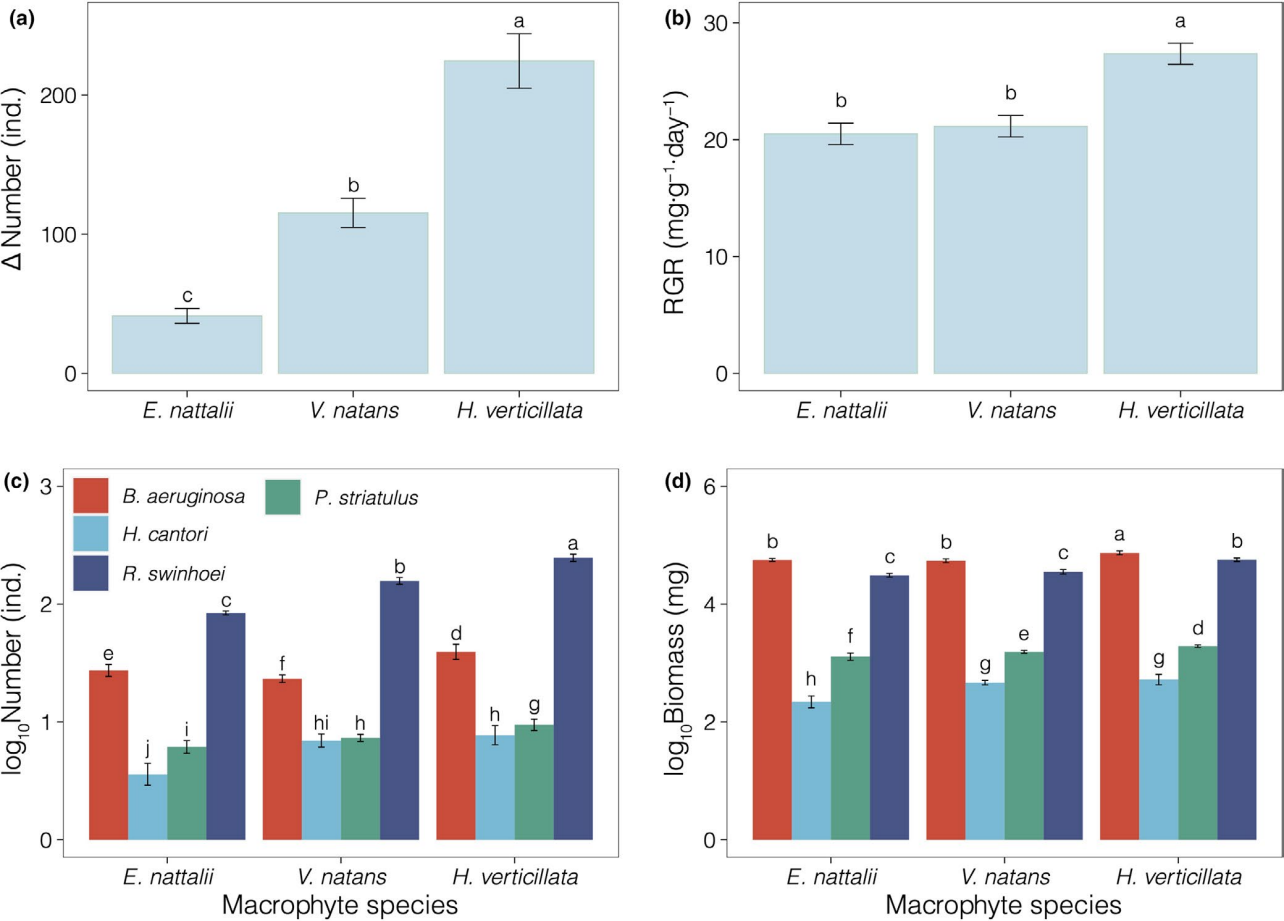
Comparison of the total snail number (a), total snail relative growth rate (b), and the number (c) and biomass (d) of four snail species (i.e., *B. aeruginosa*, *H*. *cantori*, *P*. *striatulus*, and *R*. *swinhoei*) in the presence of different macrophyte species during the experiment. Values represent the mean ± SD; means with the different letters are significantly different at *p* < .05 (LSD test)

The number of individuals and RGR of the snail species were markedly affected by the macrophyte species (Table 4, *p* < .001). The increase in number and RGR was greatest during the experiment in the presence of *H*. *verticillata* (Figure 2a,b).

**TABLE 4:** Effect of macrophytes on snail number (Δ Number) and snail relative growth rate (RGR) during the experiment using one‐way ANOVA (values in bold are below the significance level of .05)

	Δ Number		RGR	
	*F_2,15_*	*p*	*F_2,15_*	*p*
Macrophyte	293.4	**<.001**	103.1	**<.0001**

The biomass and number of the four species of snails (i.e., *B. aeruginosa*, *H*. *cantori*, *P*. *striatulus*, and *R*. *swinhoei*) were notably affected by macrophyte and snail species identity (Table 5, *p* < .001). Significant interactions between macrophytes and snail species were observed for four snail species (Table 5, *p* < .001). *R*. *swinhoei* and *B. aeruginosa* had the greatest number of individuals and biomass in the presence of all macrophyte species, with the maximum value occurring in the presence of *H*. *verticillata* (Figure 2c,d).

**TABLE 5:** Effects of macrophytes on the number and biomass of four snail species during the experiment using two‐way ANOVA (values in bold are below the significance level of .05)

	Biomass			Number		
	df	*F*	*p*	df	*F*	*p*
Macrophyte	2	132.92	**<.001**	2	172.22	**<.001**
Species	3	8258.96	**<.001**	3	2631.18	**<.001**
Macrophyte × species	6	15.47	**<.001**	6	19.69	**<.001**

### 3.4 Phytoplankton and epiphytic algae

**FIGURE 3:**
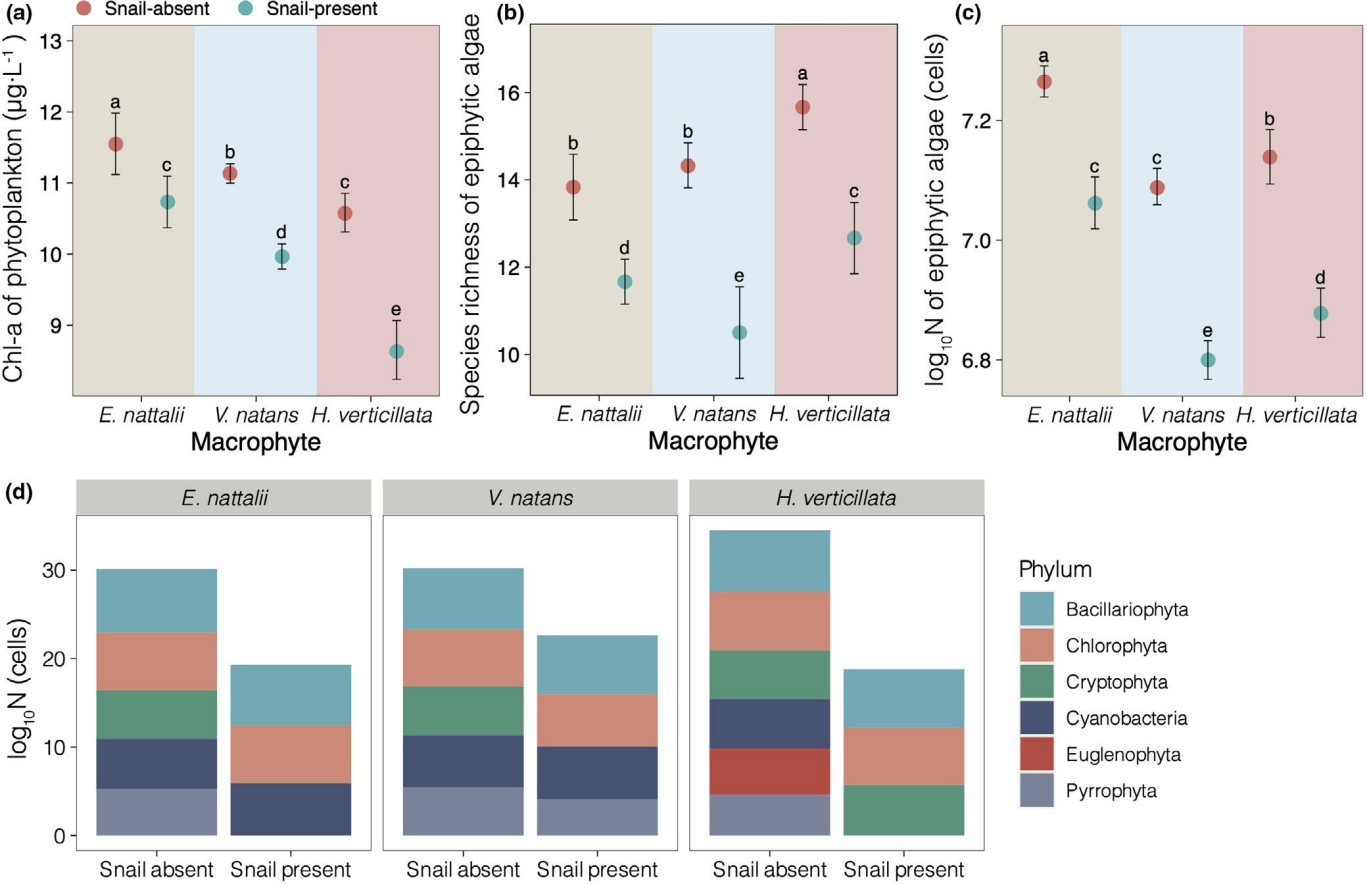
Comparison of water chlorophyll a concentration (a), epiphytic algal species richness (b), epiphytic algal abundance (c), the abundance of six phyla of epiphytic algae, and (d) in the presence of macrophytes and snail grazing during the experiment. Values represent the mean ± SD; means with the different letters are significantly different at *p* < .05 (LSD test)

The Chl‐*a* concentration was markedly affected by the submerged macrophyte species and snail presence (Table 5, *p* < .001), and there was a significant interaction between macrophyte species and the presence of snails in terms of the Chl‐*a* concentration (Table 5, *p* = .001). The Chl‐*a* concentrations in the presence of *V*. *natans* were significantly lower than those in the presence of *E*. *nuttallii* and *H*. *verticillata* in both the presence and absence of snails (Figure 3a). The presence of snails consistently led to significantly lower Chl‐*a* concentrations in association with the three macrophyte species (Figure 3a).

Snails significantly decreased the richness and abundance of epiphytic algae (Table 5, *p* < .001; Figure 3b,c), and macrophyte species markedly affected the richness and abundance of epiphytic algae (Table 5, *p* < .001). Macrophyte species and snail treatments had significant interactive effects on epiphytic algal richness and abundance (Table 5, *p* < .001). The epiphytic algal richness in the presence of *H*. *verticillata* was significantly greater than that in the presence of *E*. *nuttallii* and *V*. *natans* in both the presence and absence of snails (Figure 3b). The epiphytic algal abundance in the presence of *V*. *natans* was significantly lower than that in the presence of *E*. *nuttallii* and *H*. *verticillata* when snails were both present and absent (Figure 3c). A total of 35 epiphytic algae species belonging to six phyla were identified on three submerged macrophytes in 36 aquariums. Eleven genera of diatoms, 17 genera of green algae, four genera of blue–green algae, and one genus of cryptomonads, euglenoids, and dinoflagellates were identified (Table S1; Figure S2). Diatoms and green algae accounted for most of the epiphytic algae (Figure 3d). When snails were present, the abundance of diatoms and green algae tended to decrease (Figure 3d; Table 6).

**TABLE 6:** Effects of macrophytes and snail grazing on the chlorophyll *a* concentration in water and epiphytic algal richness and abundance during the experiment using two‐way ANOVA (values in bold are below the significance level of .05)

	Chl‐*a*			Abundance		Richness	
	df	*F*	*p*	*F*	*p*	*F*	*p*
Macrophyte	2	69.47	**<.001**	441.1	**<.001**	110.53	**<.001**
Snail grazing	1	150.28	**<.001**	775.82	**<.001**	553.47	**<.001**
Macrophyte × snail grazing	2	9.56	.**001**	11.41	**<.001**	20.18	**<.001**

### 3.5 Snail–macrophyte–epiphytic algae relationship

**FIGURE 4:**
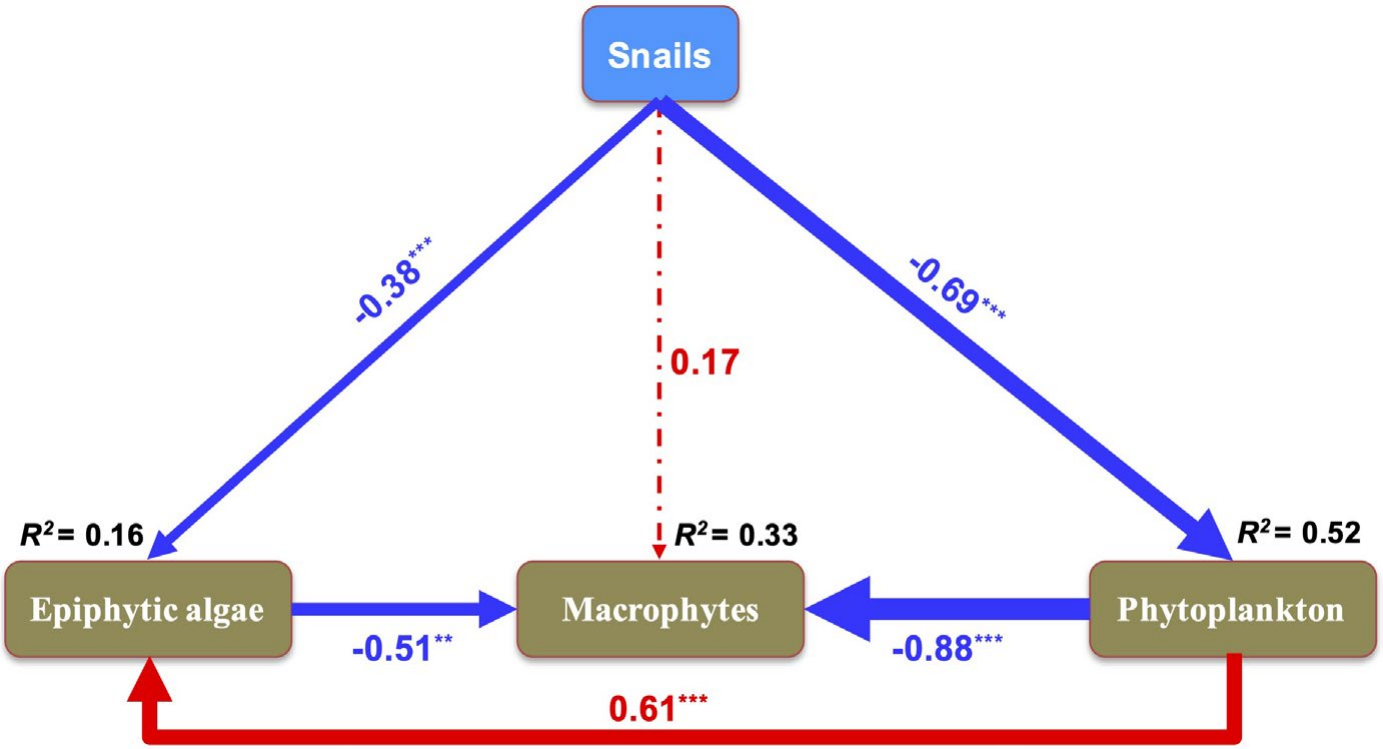
Structural equation model of the relationship between snails, macrophytes, epiphytic algae, and phytoplankton. Red and blue arrows represent significant positive and negative pathways, respectively. Arrow width is proportional to the strength of the relationship, and solid and dotted lines represent significant and nonsignificant pathways, respectively. Numbers indicate the standard path coefficients (*C*). *χ* ^2^ = 1.42, *p* < .001; *GFI* = 1; *RMSEA* < 0.001. Significance levels are indicated by asterisks: ***p < .001, **p < .01, *p < .05. (*n* = 36)

Snails (RGR) had a significantly negative effect on epiphytic algae (abundance, standard path coefficient: *C* = −0.38, *p* < .001) and phytoplankton (Chl‐*a*, *C* = −0.69, *p* < .001; Figure 4) and a nonsignificant positive effect on macrophytes (RGR, *C* = 0.17, *p* = .053; Figure 4). Epiphytic algae (*C* = −0.51, *p* = .007) and phytoplankton (*C* = −0.88, *p* < .001) both had significant negative effects on macrophytes (Figure 4). Phytoplankton had a significant positive effect on the epiphytic algae (*C* = 0.61, *p* < .001; Figure 4). The model shows that snails affect macrophytes by reducing epiphytic algae and phytoplankton (biomass decrease) to improve the biomass of macrophytes.

**FIGURE 5:**
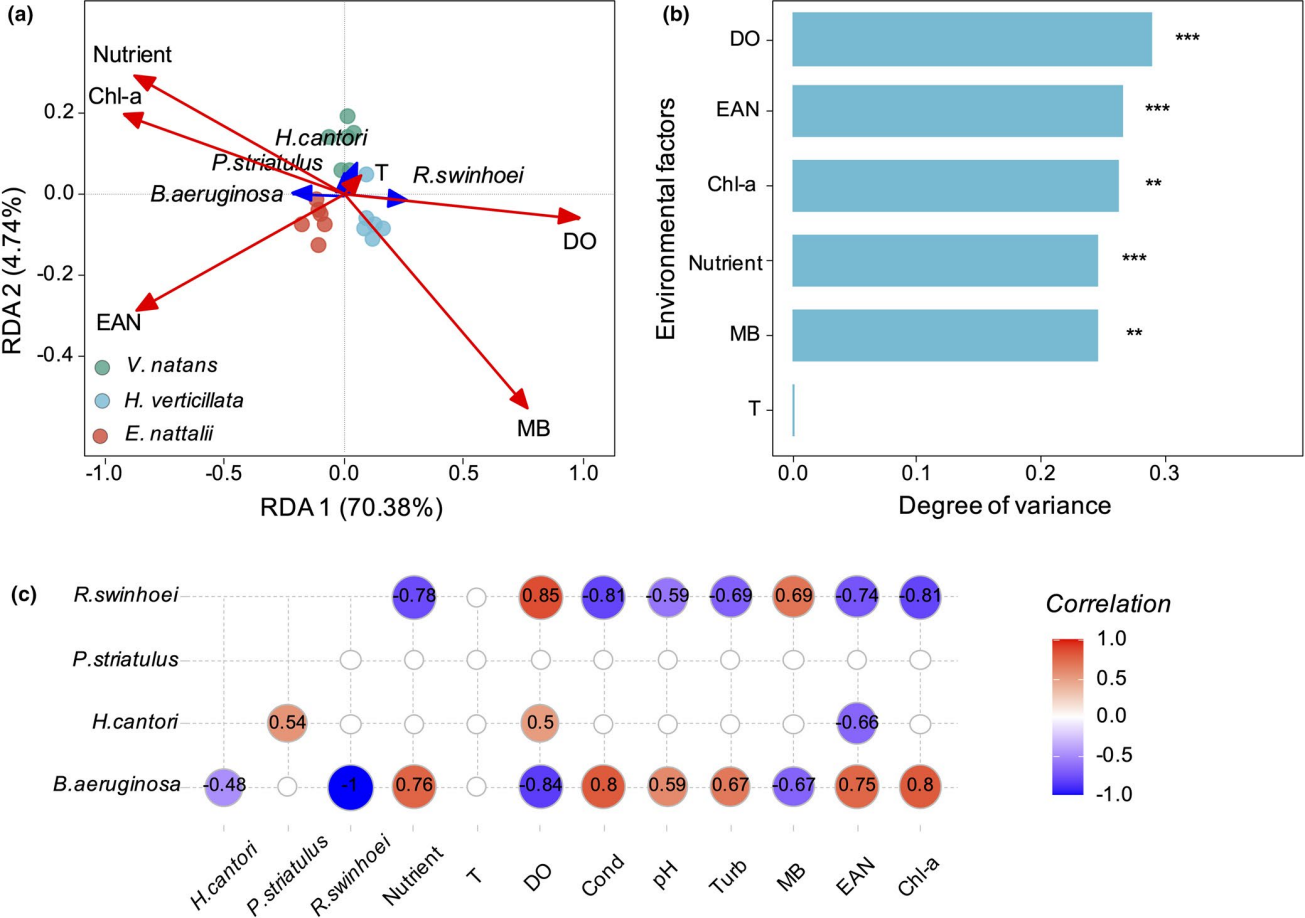
Relationships among snail species and environmental factors based on RDA. The figure was based on the snail biomass and environmental factor data (i.e., macrophyte biomass‐MB, epiphytic algae abundance‐EAN, T, DO, nutrient, and Chl‐*a*, *n* = 18). (a) Shows the RDA plot of the snail species, environmental factors, and samples. Environmental variables are represented with red arrows; vectors represent four snail species with blue arrows; samples are represented with a filled circle. (b) Shows the effects of the environmental factors on the snail community structure. Significance levels are indicated by asterisks: ***p < .001, **p < .01, *p < .05. (c) Shows the correlation among the four snail species and environmental factors. The snail species data are based on the Hellinger transformation of biomass. Correlation coefficients with *p* values below .05 are shown

The two main axes of the RDA indicate a significant relationship between environmental variables and the biomasses of four species of snails (explaining 75.84% of the total variance, *p* = .001; Figure 5a). Snail community structure was significantly affected by DO (degree of variance: *R* ^2^ = .29, *p* < .001), nutrients (*R* ^2^ = .25, *p* < .001), epiphytic algae abundance (*R* ^2^ = .27, *p* < .001), phytoplankton biomass (*R* ^2^ = .26, *p* = .002), and macrophyte biomass (*R* ^2^ = .25, *p* = .002; Figure 5b). The biomass of *B. aeruginosa* was significantly positively correlated with nutrient (correlation coefficient: *R* = .76, *p* < .001), epiphytic algae abundance (*R* = .75, *p* < .001), and Chl‐*a* (*R* = .80, *p* < .001) and significantly negatively correlated with macrophyte biomass (*R* = −.67, *p* = .002; Figure 5a,c). The biomass of *R*. *swinhoei* was significantly positively correlated with the macrophyte biomass (*R* = .69, *p* = .001) and negatively correlated with nutrient (*R* = −.78, *p* < .001), epiphytic algae abundance (*R* = −.74, *p* < .001), and Chl‐*a* (*R* = −.81, *p* < .001; Figure 5a,c). The biomass of *H*. *cantori* was significantly negatively correlated with epiphytic algae abundance (*R* = −.66, *p* = .003; Figure 5a,c). There was no significant correlation between *P*. *striatulus* and any one of the environmental factors (*p* > .05, Figure 5a,c).

## 4 DISCUSSION

Snails positively affected submerged macrophyte growth and development by increasing biomass, as demonstrated in both simulation experiments and field investigations (Li et al., 2008; Mormul et al., 2018; Yang et al., 2020). We found that the presence of snails significantly reduced the biomass of epiphytic algae and phytoplankton (Figure 3). Earlier studies showed that shading by epiphytic algae and phytoplankton might limit the growth of submerged macrophytes (Arthaud et al., 2012; Song et al., 2017; Tóth, 2013); hence, grazing by snails should favor macrophyte growth by decreasing the competition for light among epiphytic algae, phytoplankton, and submerged macrophytes (Hidding et al., 2016; Yang et al., 2020). SEM results showed that snails decreased the epiphytic algae (*C* = −0.38, *p* < .001) and phytoplankton (*C* = −0.69, *p* < .001) biomass through the improvement of macrophyte RGR (Figure 4). In addition, the pathway from the snail to macrophytes was nonsignificant (Figure 4), which indicates that the changes in snails cannot directly explain the variation in macrophytes. These results further demonstrate that snail communities have indirect positive effects on submerged macrophyte growth through the removal of epiphytic algae and phytoplankton (Mormul et al., 2018).

On the other hand, in the snail‐present treatment, the nutrients in the water were significantly lower than those in the snail‐absent treatment (Table 2). The snail community might eliminate the competition between epiphytic algae and phytoplankton with macrophytes for resources (light and nutrients), and a large amount of nutrients in the water column are absorbed by macrophytes to supply their growth and reproduction (Cao et al., 2018; Kuiper et al., 2017; Li et al., 2019). Furthermore, the increase in macrophyte biomass could inhibit epiphytic algae and phytoplankton by enhancing competition for resources (light and nutrients) (Jones et al., 2000; Kuiper et al., 2017). We also found that increasing macrophyte biomass could increase the species richness of epiphytic algae (*R* = .43, *p* = .008; Figure S3), possibly by providing more diverse and heterogeneous habitats for epiphytic algae or decreasing intraspecific competition in the epiphytic algal community (Celewicz‐Gołdyn & Kuczyńska‐Kippen, 2017; Lv et al., 2019; Toporowska et al., 2008).

In this experiment, both the number and biomass of the snail communities were greatest on *H*. *verticillata* (Figure 2). The architectural complexity of *H*. *verticillata* and *E*. *nuttallii* was greater than that of *V*. *natans*. This result suggests that macrophytes with relatively complex architecture (e.g., *H*. *verticillata*) might provide more habitats and spatial niches for snail communities (Mcabendroth et al., 2010). *H*. *verticillata* and *E*. *nuttallii* are members of Hydrocharitaceae and have similar leaf shapes, while the number and biomass of the snail communities on *E*. *nuttallii* were lower than those on *H*. *verticillate* (Figure 2). Although the leaf structure of *E*. *nuttallii* is more complex than that of *V*. *natans*, while the number and biomass of the snail communities on *E*. *nuttallii* were lower than those on *V*. *natans* in this study (Figure 2). This possibly occurred because *E*. *nuttallii* is an exotic species (Xie et al., 2010; Xiong et al., 2008). Native predators have gradually adapted to the defence strategies of native plants over long‐term coevolution, while they are naive to the defence strategies of foreign plants and thus prefer to feed on native plants (Keane & Crawley, 2002; Xiong et al., 2008). Native macrophytes have a long history of coevolution with native snails, which could help snails quickly adapt to habitats containing native macrophytes. On the other hand, the richness and abundance of epiphytic algae on *H*. *verticillata* (native) was significantly greater than that on *E*. *nuttallii* (exotic); accordingly, *H*. *verticillata* could provide a greater source of food for snails.

The dominant species in the snail communities were *B. aeruginosa* (58.95% of biomass on average) and *R*. *swinhoei* (78.84% of number on average) in terms of biomass and number, respectively (Figure 2). Together, *B. aeruginosa* and *R*. *swinhoei* contributed 98.09% of the total biomass on average. The biomass of *B. aeruginosa* was significantly positively correlated with epiphytic algae and phytoplankton (Figure 5a,c), namely, epiphytic algae and phytoplankton were the main food sources for *B. aeruginosa* (Han et al., 2010; Li et al., 2008; Zhu et al., 2013). The biomass of *R*. *swinhoei* was significantly positively correlated with the macrophytes in this study (Figure 5a,c), which indicates that *R*. *swinhoei* mainly fed on submerged macrophytes (Li et al., 2006; Li et al., 2009; Yang et al., 2020). Furthermore, we observed the *R*. *swinhoei* scraped the surface of the submerged macrophytes (Figure S1D,E), which suggested that the *R*. *swinhoei* might graze submerged macrophytes. *R*. *swinhoei* has a large and dense radula that makes it easy to scrape and feed on the plant tissues (Xiong et al., 2008) and easily feeds on algae. Previous studies also verified that *R*. *swinhoei* feeds on macrophytes, periphytons were found to be the main food source for this species (Li et al., 2008). Previous studies verified that *B. aeruginosa* feeds only on algae and scrap (Li et al., 2019), mainly because its radula is small enough not to damage plant tissue. First, we hypothesized that the greater the food supply was, the greater the biomass of the snails. Second, as the correlation matrix shows, the biomass of *B. aeruginosa* was significantly positively correlated with epiphytic algae and phytoplankton, and the biomass of *R*. *swinhoei* was significantly positively correlated with macrophytes (Figure 5c). According to the above, we concluded that *B. aeruginosa* mainly feeds on algae and that *R*. *swinhoei* mainly feeds on macrophytes. Competition has been identified as underlying niche divergence (Hardin, 1960); when predators have the same niche and multiple food sources, competition drives them to change their feeding preferences to achieve coexistence (Kolsch & Kubiak, 2011; Zaret & Rand, 1971). Consequently, competition drives snails to change their grazing preferences to achieve coexistence.

## 5 CONCLUSION

Snail communities can reduce the biomass of phytoplankton and epiphytic algae and thereby enhance the growth of submerged macrophytes. Macrophytes with complex architecture support more snails and epiphytic algae, and snails prefer to feed on native plants. Competition drives snails to change their grazing preferences to achieve coexistence.

## CONFLICT OF INTEREST

The authors declare that they have no conflict of interest.

## AUTHOR CONTRIBUTION

**Tian Lv:** Conceptualization (equal); Data curation (lead); Formal analysis (lead); Investigation (lead); Methodology (lead); Project administration (lead); Resources (equal); Supervision (equal); Validation (equal); Visualization (lead); Writing – original draft (lead); Writing – review & editing (lead). **Xin Guan:** Conceptualization (equal); Data curation (lead); Formal analysis (equal); Investigation (lead); Methodology (lead); Project administration (lead); Resources (lead); Validation (equal); Visualization (equal); Writing – original draft (equal); Writing – review & editing (equal). **Shufeng Fan:** Conceptualization (lead); Resources (equal); Writing – original draft (equal); Writing – review & editing (equal). **Chen Han:** Data curation (equal); Investigation (equal); Visualization (equal); Writing – original draft (equal). **Zhongyao Gao:** Data curation (equal); Formal analysis (equal); Investigation (equal); Writing – original draft (equal). **Chunhua Liu:** Conceptualization (lead); Funding acquisition (lead); Resources (equal); Supervision (lead); Validation (lead); Writing – original draft (equal); Writing – review & editing (equal).

### OPEN RESEARCH BADGES

This article has earned an Open Data Badge for making publicly available the digitally‐shareable data necessary to reproduce the reported results. The data is available at [https://doi.org/10.5061/dryad.dz08kprxf].

## Supporting information

Fig S1

Fig S2

Fig S3

Table S1‐S4

## Data Availability

All data used in the production of this article are available via Dryad: https://doi.org/10.5061/dryad.dz08kprxf.
