# Supplementary figures and images for: Snail communities increase submerged macrophyte growth by grazing epiphytic algae and phytoplankton in a mesocosm experiment
Source: Ecol Evol. 2022 Feb 14;12(2):e8615. doi: 10.1002/ece3.8615 (PMC8843764; doi:10.1002/ece3.8615)

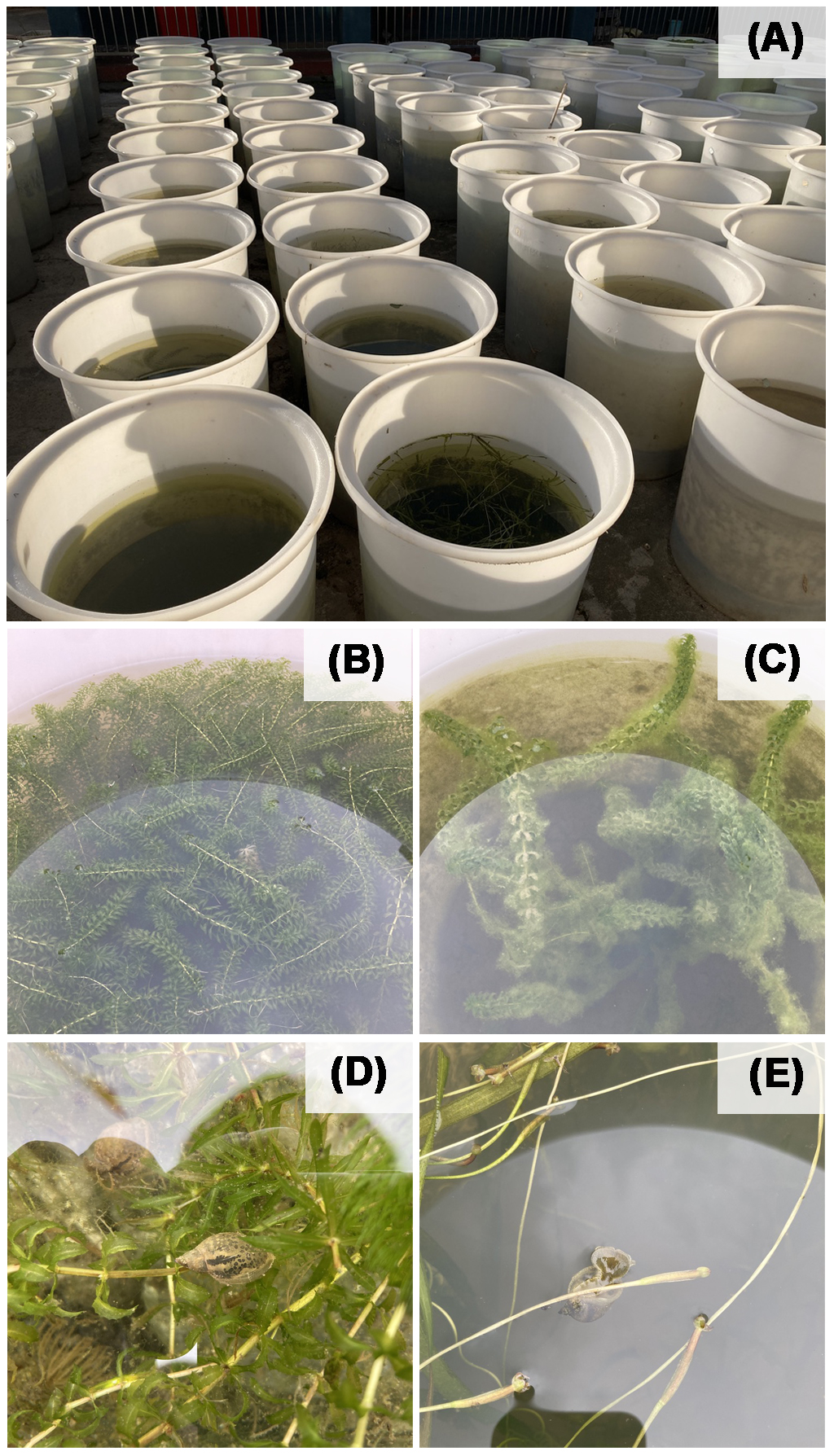

Supplement: Supplementary file 1 — Fig S1 [file ECE3-12-e8615-s002.jpg]

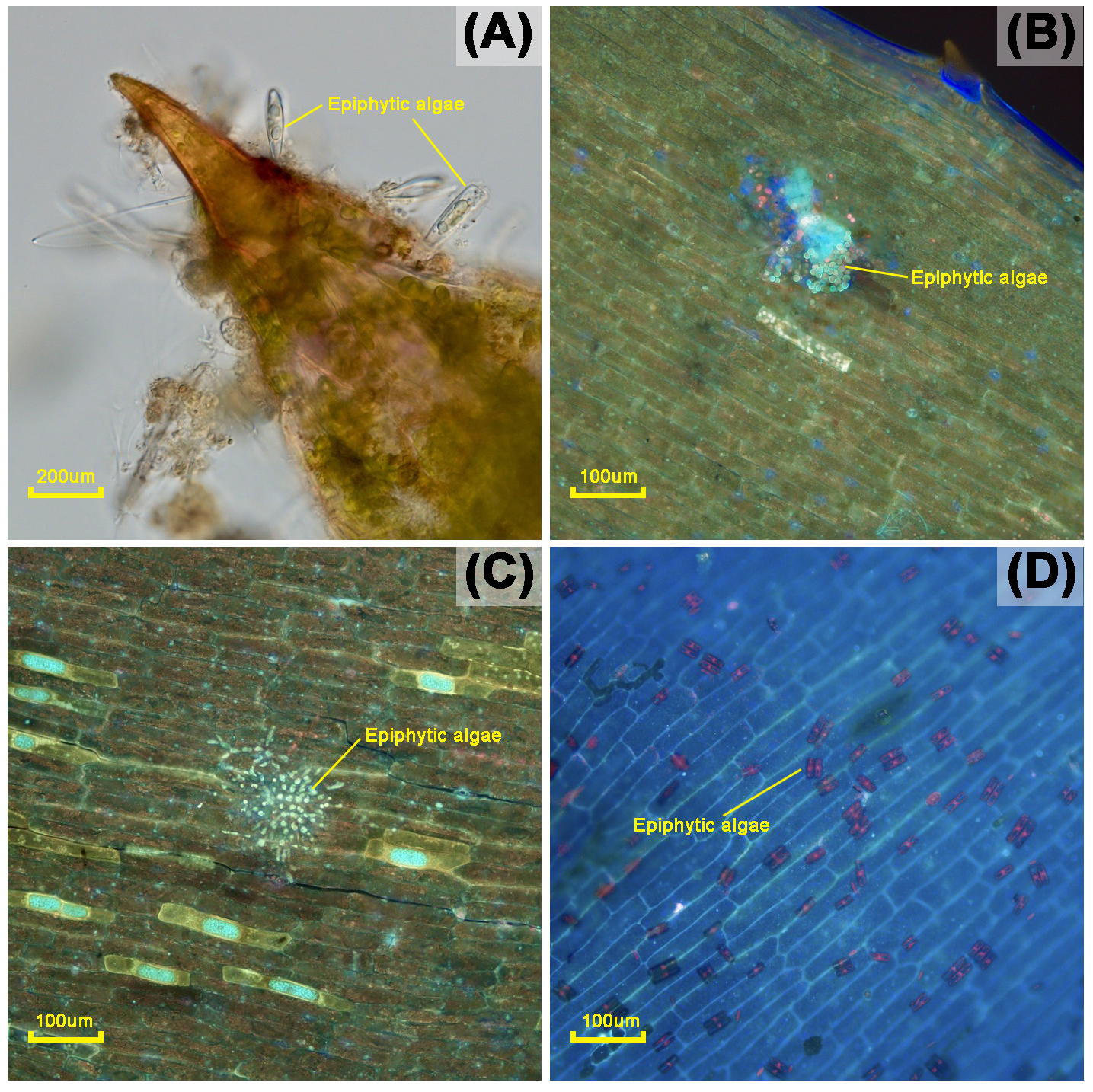

Supplement: Supplementary file 2 — Fig S2 [file ECE3-12-e8615-s004.jpg]

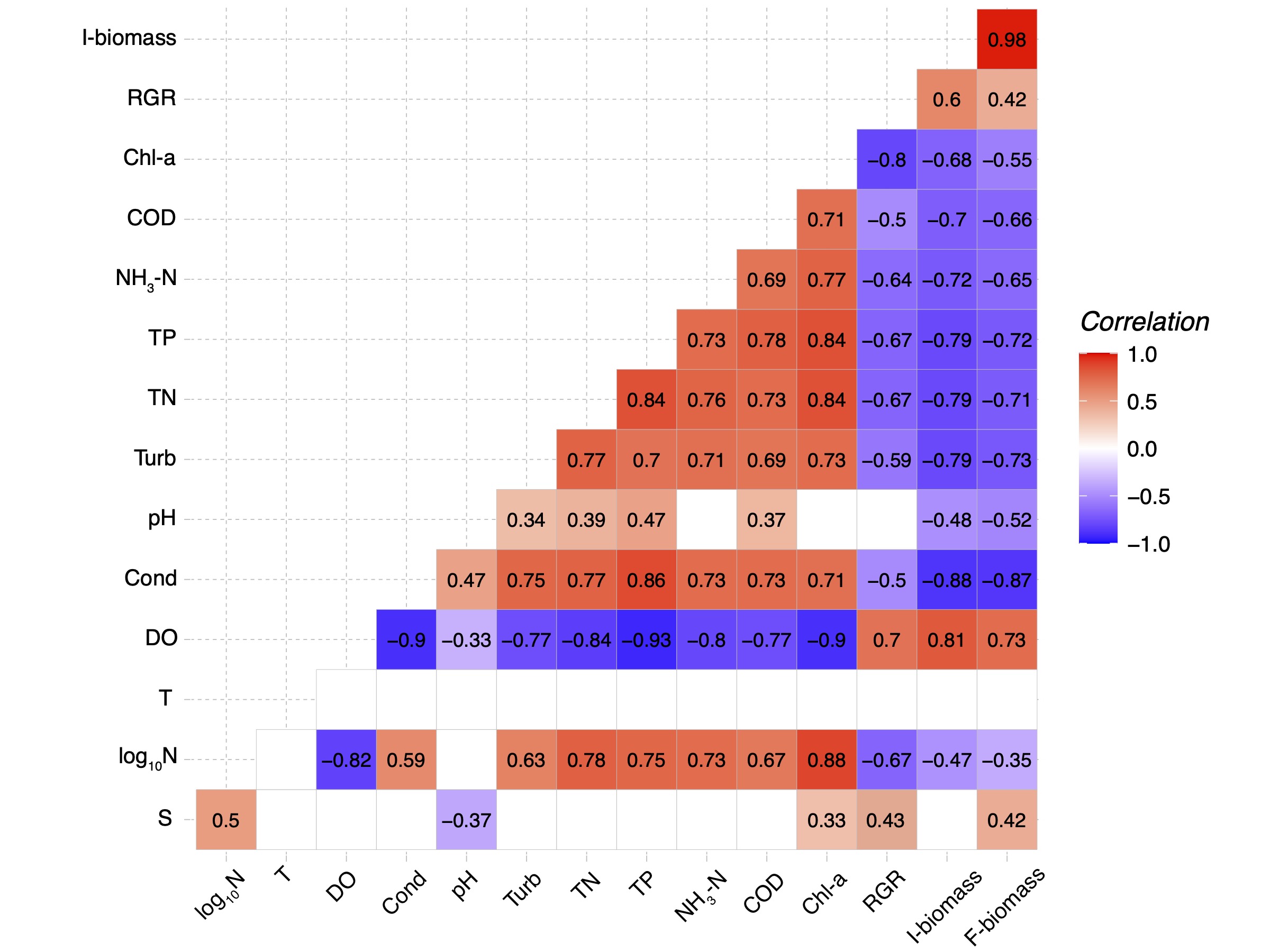

Supplement: Supplementary file 3 — Fig S3 [file ECE3-12-e8615-s003.jpg]
