# Supplementary material for: Snail communities increase submerged macrophyte growth by grazing epiphytic algae and phytoplankton in a mesocosm experiment
Source: Ecol Evol. 2022 Feb 14;12(2):e8615. doi: 10.1002/ece3.8615 (PMC8843764; doi:10.1002/ece3.8615)
Supplement: Supplementary file 4 — Table S1‐S4 [file ECE3-12-e8615-s001.docx]

# Appendix

**Table S1** The species list of epiphytic algae in this study.

| **ID** | **species** | **Phylum** | **Mcnaughton dominance** |
| --- | --- | --- | --- |
| BA01 | *Achnanthes exigua* | Bacillariophyta | 0.568 |
| BA02 | *Rhopalodia gibba* | Bacillariophyta | 0.006 |
| BA03 | *Nitzschia amphibia* | Bacillariophyta | 0.000 |
| BA04 | *Cymbella cistula* | Bacillariophyta | 0.001 |
| BA05 | *Gomphonema constrictum* var*. capitatum* | Bacillariophyta | 0.003 |
| BA06 | *Gomphonema gracile* | Bacillariophyta | 0.006 |
| BA07 | *Gomphonema subclavatum* | Bacillariophyta | 0.009 |
| BA08 | *Gomphonema parvulum* | Bacillariophyta | 0.004 |
| BA09 | *Navicula reichardtiana* | Bacillariophyta | 0.000 |
| BA10 | *Cocconeis placentula* | Bacillariophyta | 0.000 |
| BA11 | *Melosira granulata* | Bacillariophyta | 0.000 |
| CH01 | *Ankistrodesmus falcatus* | Chlorophyta | 0.006 |
| CH02 | *Ankistrodesmus spiralis* | Chlorophyta | 0.014 |
| CH03 | *Tetraedron minimum* | Chlorophyta | 0.002 |
| CH04 | *Tetraedron trigonum* | Chlorophyta | 0.000 |
| CH05 | *Pediastrum tetras* | Chlorophyta | 0.000 |
| CH06 | *Coelastrum microporum* | Chlorophyta | 0.016 |
| CH07 | *Scenedesmus quadricauda* | Chlorophyta | 0.007 |
| CH08 | *Scenedesmus dimorphus* | Chlorophyta | 0.001 |
| CH09 | *westella botryoides* | Chlorophyta | 0.000 |
| CH10 | *Cosmarium pseudobroomei* | Chlorophyta | 0.021 |
| CH11 | *Eudorina elegans* | Chlorophyta | 0.003 |
| CH12 | *Closterium leibleinii* | Chlorophyta | 0.001 |
| CH13 | *Cosmarium leave* | Chlorophyta | 0.000 |
| CH15 | *Cosmarium obtusatum* | Chlorophyta | 0.000 |
| CH16 | *Euastrum spinulosum* | Chlorophyta | 0.016 |
| CH17 | *Staurastrum gracile* | Chlorophyta | 0.005 |
| CH18 | *Spirogyra sp.* | Chlorophyta | 0.000 |
| CR01 | *Cryptomonas ovata* | Cryptophyta | 0.006 |
| CY01 | *Oscillatoria fraca* | Cyanophyta | 0.061 |
| CY02 | *Merismopedia sinica* | Cyanophyta | 0.001 |
| CY03 | *Merismopedia punciata* | Cyanophyta | 0.000 |
| CY04 | *Oscillatoria princes* | Cyanophyta | 0.000 |
| EU01 | *Euglena viridis* | Euglenophyta | 0.001 |
| PY01 | *Peridiniopsis cunningtonii* | Pyrrophyta | 0.000 |

**Table S2** Principal component analysis (PCA) of nutrients. The correlation coefficient (*R*) and principal component scoring coefficient (*K*) between TN, TP, NH_3_-N and COD were assigned to one principal component (i.e., nutrient). Standardized loadings (pattern matrix) based upon: Mean item complexity = 1; Test of the hypothesis that 1 component is sufficient. The root mean square of the residuals (RMSR) is 0.02, with an empirical chi square of 0.09 and prob < 0.95. Fit based upon off diagonal values = 1.

|  | *R* | *K* | *h^2^* | *u^2^* | com |
| --- | --- | --- | --- | --- | --- |
| TN | 0.99 | 0.26 | 0.97 | 0.03 | 1 |
| TP | 0.97 | 0.26 | 0.95 | 0.05 | 1 |
| NH_3_-N | 0.98 | 0.26 | 0.95 | 0.049 | 1 |
| COD | 0.96 | 0.25 | 0.92 | 0.08 | 1 |

**Table S3** The effect of snails on macrophytes based on a structural equation model (SEM). Model: sem(RGR _Mac_ ~ RGR _Snail_ + Chl-a + EAN, EAN ~ RGR _snail_ + Chl-a, Chl-a ~ RGR _Snail_); RGR _Mac_ was the relative growth rate of macrophytes, RGR _Snail_ was the relative growth rate of snails, EAN was the epiphytic algae abundance, Chl-a was the Chl-a concentrations of phytoplankton. **A** was result of model fit index. **B** was results of regressions and variances in the SEM.

**Table S3 (A)** Parameters and fit index of model.

| Parameters or Fit index | Value |
| --- | --- |
| Number of model parameters | 9 |
| Number of observations | 36 |
| Degrees of freedom (*df*) | 6 |
| *P* value | <0.001 |
| *χ^2^* | 1.42 |
| Goodness-of-Fit Index (GFI) | 1 |
| Comparative Fit Index (CFI) | 1 |
| Tucker–Lewis Index (TLI) | 1 |
| Root Mean Square Error of Approximation (RMSEA) | <0.001 |

**Table S3 (B)** The results of regression between explanatory variable (*X*) and response variable (*Y*) in the SEM. Variances of response variable in the SEM.

| Model | *Y* | *X* | Estimate | Std.Err | Z value | *P*(>\|z\|) | Std.lv | Std.all |
| --- | --- | --- | --- | --- | --- | --- | --- | --- |
| Regressions | RGR _Mac_ | RGR _Snail_ | 0.036 | 0.012 | 0.948 | 0.053 | 0.036 | 0.174 |
|  |  | EAN | -0.163 | 0.077 | -2.125 | 0.007 | -0.163 | -0.506 |
|  |  | Chl-a | -1.066 | 0.237 | -4.491 | <0.001 | -1.066 | -0.881 |
|  | EAN | RGR _Snail_ | -0.089 | 0.022 | -4.15 | <0.001 | -0.089 | -0.384 |
|  |  | Chl-a | 2.283 | 0.348 | 6.563 | <0.001 | 2.283 | 0.607 |
|  | Chl-a | RGR _Snail_ | -0.043 | 0.007 | -5.725 | <0.001 | -0.043 | -0.69 |
| Model | | Parameters | Estimate | Std.Err | z value | *P*(>\|z\|) | Std.lv | Variances |
| Variances | | RGR _Mac_ | 0.001 | 0 | 4.243 | <0.001 | 0.001 | 0.33 |
|  |  | EAN | 0.004 | 0.001 | 4.243 | <0.001 | 0.004 | 0.16 |
|  |  | Chl-a | 0.001 | 0 | 4.243 | <0.001 | 0.001 | 0.52 |

**Table S4** The relationships among snail species and environmental factors based on a redundancy analysis (RDA). Model: rda (Snail_hell_ ~ MB + Chl-a + EAN + T + DO + Nutrient, env); Snail_hell_ was the matrix of Hellinger-transformed snail biomass, env was the matrix of environmental factors (including MB, Chl-a, EAN, T, DO and nutrient; where MB was macrophyte biomass, EAN was the epiphytic algae abundance, and Chl-a was the Chl-a concentration of phytoplankton). **A** and **B** was the results of constrained. **C** was permutation test for constrained eigenvalues. **D** was permutation test for environmental factors.

**Table S4 (A)** Proportion of constrained and unconstrained individuals. Note: Inertia is variance

|  | Inertia | Proportion | Rank |
| --- | --- | --- | --- |
| Total | 0.0017502 | 1 |  |
| Constrained | 0.0013274 | 0.7584463 | 4 |
| Unconstrained | 0.0004228 | 0.2415537 | 4 |

**Table S4 (B)** Eigenvalues for constrained and unconstrained axes.

| Eigenvalues for constrained axes | RDA1 | RDA2 | RDA3 | RDA4 |
| --- | --- | --- | --- | --- |
|  | 0.0012317 | 0.000083 | 0.0000127 | 0 |
| Eigenvalues for unconstrained axes | PC1 | PC2 | PC3 | PC4 |
|  | 3.95E-04 | 2.14E-05 | 5.90E-06 | 4.00E-07 |

**Table S4 (C)** Permutation test for constrained eigenvalues. Monte Carlo permutation test, reduced model, 499 permutations. Note: Inertia is variance.

|  | *Df* | Inertia | *F* | *P* (>*F*) |
| --- | --- | --- | --- | --- |
| Model | 6 | 0.00132742 | 5.7564 | 0.001 |
| Residual | 11 | 0.00042276 |  |  |

**Table S4 (D)** Permutation test for environmental factors. Monte Carlo permutation test, reduced model, 499 permutations.

|  | Variance | P(>r) |
| --- | --- | --- |
| Nutrient | 0.248 | 0.001 |
| T | 0.002 | 0.951 |
| DO | 0.291 | 0.001 |
| MB | 0.248 | 0.002 |
| EAN | 0.267 | 0.001 |
| Chl-a | 0.263 | 0.002 |

**
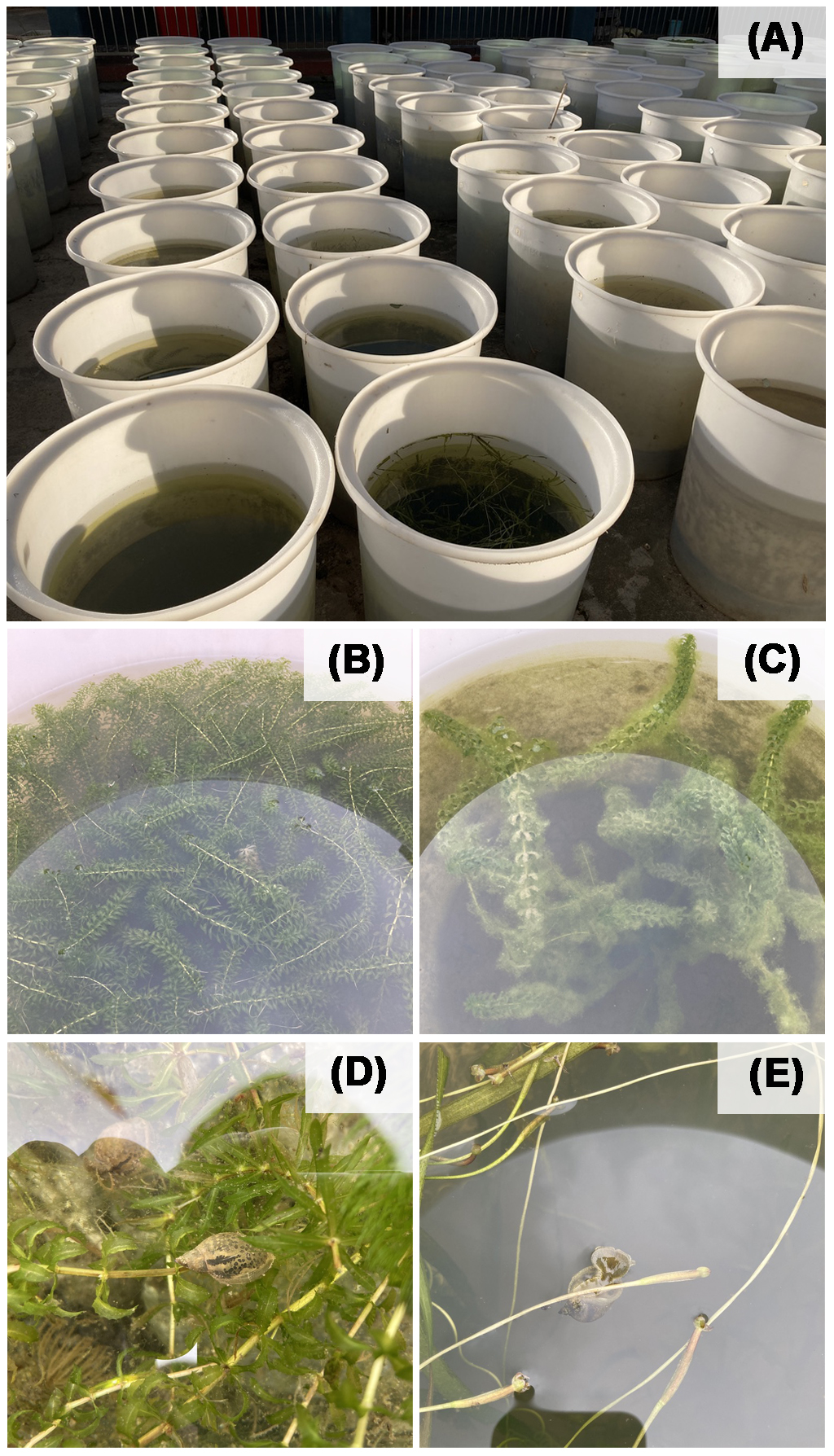
**

**Figure S1 (A)** was photo of containers and experimental site. **(B)** and **(C)** show photographs of snails present and absent treatment, respectively. **(D)** and **(E)** shows snails on *H. verticillata* and *V. natans*, respectively.

**
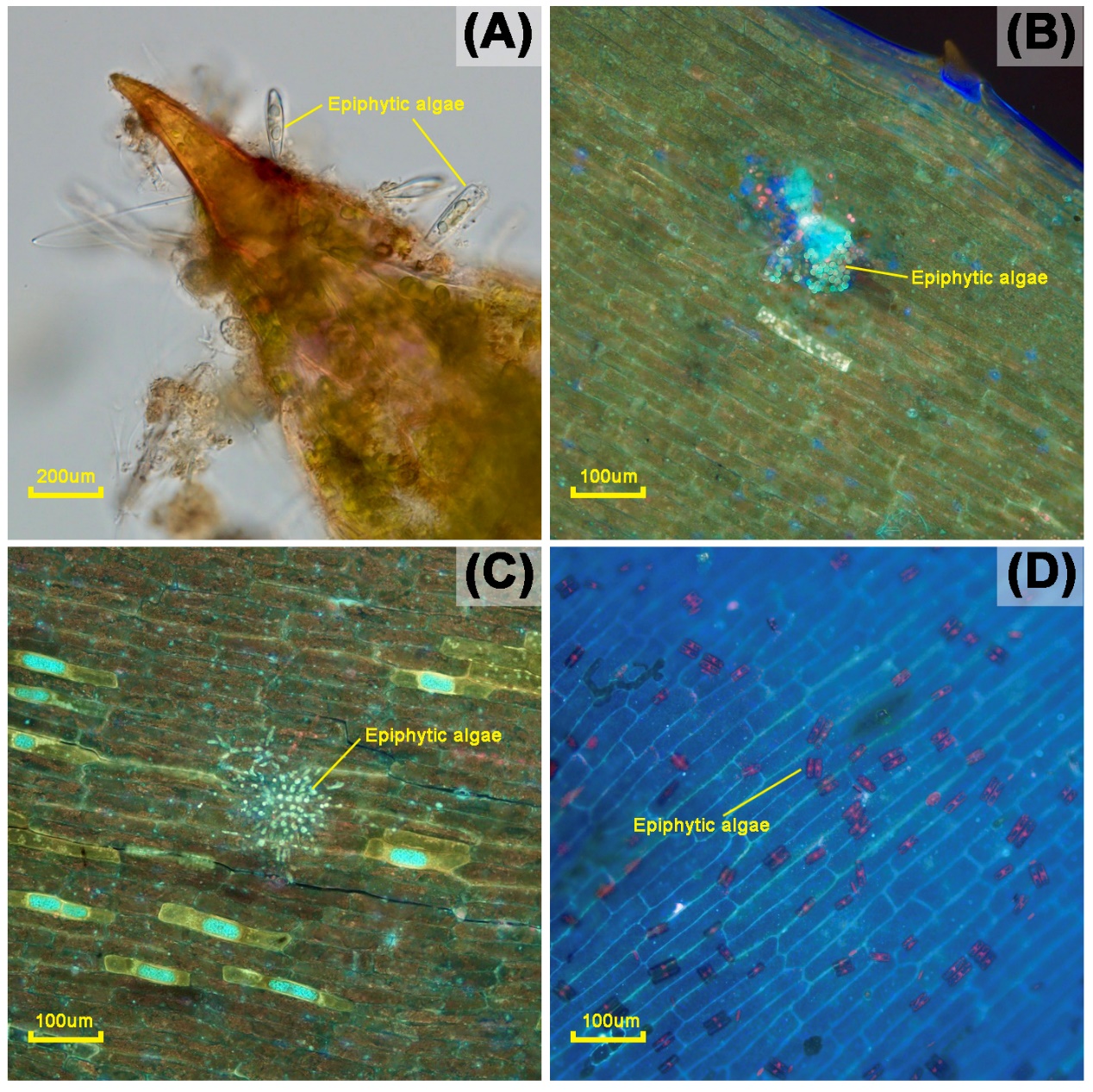
**

**Figure S2** Microscope observations of epiphytic algae on the leaves of submerged macrophytes. **(A)** shows epiphytic algae on the leaf of *H. verticillata* in the contrast phase field by a fluorescent contrast phase microscope (BX53, OLYMPUS, Japan). **(B), (C)** and **(D)** show epiphytic algae on the leaves of *H. verticillata*, *V. natans* and *E. nuttallii* in the fluorescent field by a fluorescent contrast phase microscope (BX53, OLYMPUS, Japan), respectively; algae cells show red or yellow fluorescence.

**
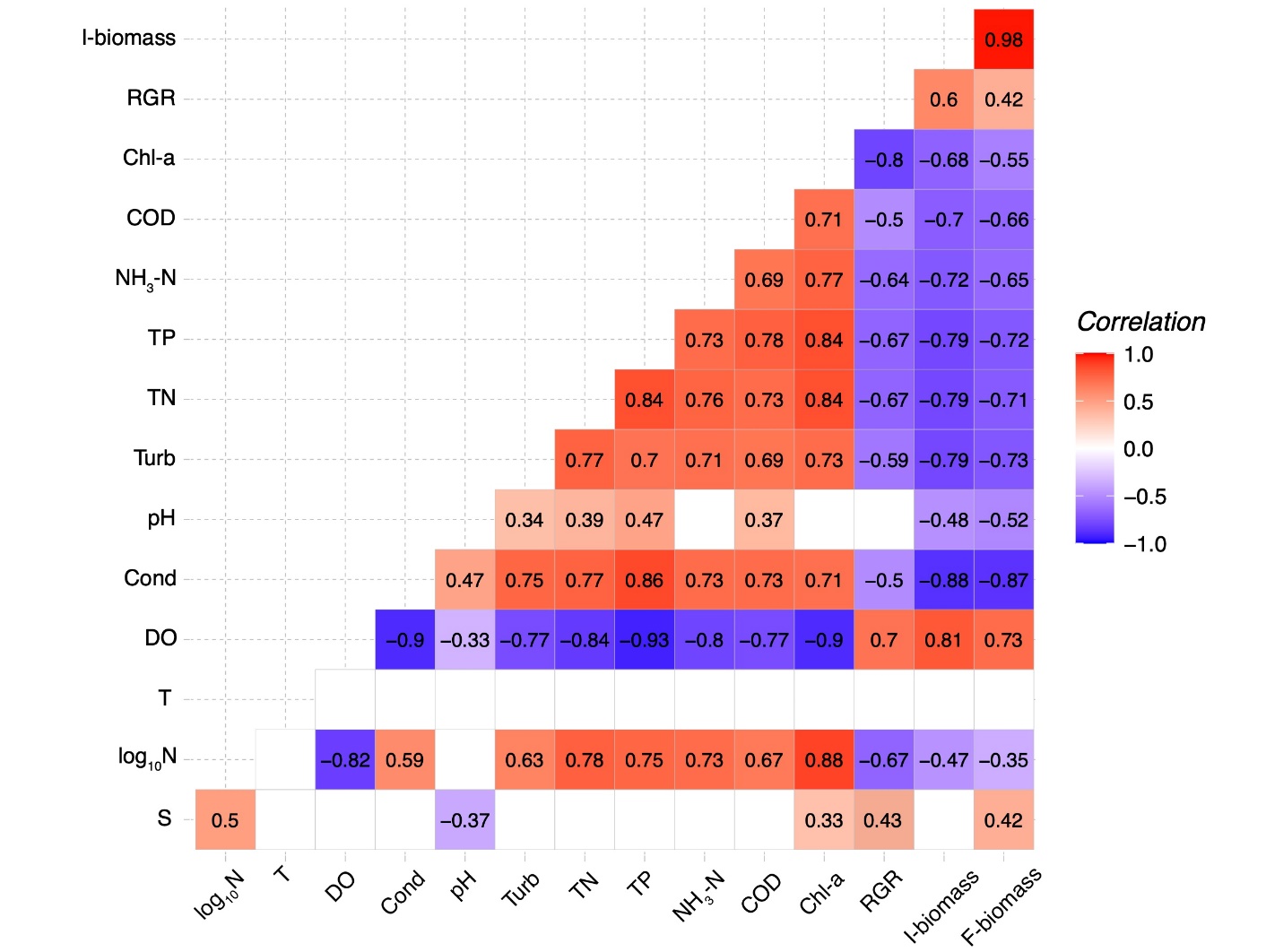
**

**Figure S3** The correlation among epiphytic algal traits (i.e., abundance (N) and species richness (S)), macrophyte traits (i.e., initial biomass (I-biomass), final biomass (F-biomass), and relative growth rate (RGR)), phytoplankton biomass (Chl-a) and environmental factors. Correlation coefficients with *P* values below 0.05 are shown (n = 36).
